# Mediastinitis and septic shock complicating spontaneous esophageal rupture “Boerhaave’s syndrome”: a case report

**DOI:** 10.1186/s12245-024-00642-0

**Published:** 2024-05-08

**Authors:** Said Kortli, Hery Andrianjafy

**Affiliations:** Emergency Department, Arpajon General Hospital, Arpajon, France

## Abstract

Boerhaave’s syndrome, also known as spontaneous esophageal rupture, is a rare but life-threatening condition characterized by a tear in the esophagus. It is most commonly caused by a sudden increase in intraesophageal pressure, often due to severe vomiting or retching.

Early diagnosis of Boerhaave’s syndrome is crucial for improving patient outcomes. The classic triad of symptoms includes severe chest pain, vomiting, and subcutaneous emphysema (air under the skin). However, not all patients present with this triad, and the diagnosis can be challenging, especially in patients without the typical symptoms.

In this case report, we present the clinical details of a 52-year-old male patient who presented to the emergency department (ED) with severe abdominal pain and vomiting for several days. The patient had a history of chronic alcohol abuse and a recent episode of vigorous vomiting.

## Introduction

Esophageal perforation is a rare clinical entity with an estimated incidence of 3.1 per 1,000,000 per year [1]. It is most commonly caused by iatrogenic mechanisms, like endoscopy or surgery-related phenomena, or non-iatrogenic trauma. An extremely rare cause is the effort rupture of the esophagus, also known as Boerhaave’s syndrome, which accounts for 15% of esophageal perforation cases, as a consequence of a sudden increase in intraluminal esophageal pressure [2].

Boerhaave syndrome consists of spontaneous longitudinal transmural rupture of the esophagus. The syndrome is named after a German doctor, HERMAN BOERHAAVE, who first described it in 1724 [3].

It is a potentially lethal and frequently elusive medical condition which presents not only a diagnostic but also a therapeutic challenge. It is insufficiently considered in diagnostic hypotheses. Errors in diagnosis are usually caused by unawareness of its varied and atypical presentations or failure to consider its possibility in acute cardiothoracic and upper gastrointestinal conditions. Early aggressive surgical intervention in the form of open and wide mediastinal and chest drainage, with or without oesophageal repair, resection or exclusion, offers the patient the best chance of survival against this otherwise invariably fatal event [4].

All clinicians need to be aware of this lethal disease, its frequently unusual presentations and the importance of early diagnosis.

The clinical manifestation of spontaneous rupture of the esophagus depends on the rupture location. In 50% of the cases, it is manifested by Mackler’s triad: vomiting, lower thoracic pain and subcutaneous emphysema [5].

The focus of this case report is on an adult patient who experienced a spontaneous rupture of the esophagus. The significance of discussing this case lies in the rarity of the condition, the potential for misdiagnosis, and the negative impact that delayed diagnosis can have on the patient's outcome.

In this case, the patient presented with severe abdominal pain and vomiting, which raised suspicion for Boerhaave’s syndrome. The patient's history of chronic alcohol abuse and recent episode of vigorous vomiting further supported the diagnosis.

## Clinical summary—medical history

### Case presentation

A 52-year-old man with a past history of chronic alcoholism presented to the ARPAJON emergency department (ED) on July 4th with abdominal pain, acute vomiting, and a four-day history of bowel obstruction.

On examination, the patient was apyrexial.

He appeared distressed and showed signs of respiratory distress. His vital signs were unstable, with low blood pressure and a rapid heart rate, requiring an immediate volume expansion with crystalloids.

The Thoraco-abdominal examination revealed an acute abdomen, reduced air entry at the right base of his chest, and a left apical emphysema.

### Investigations

Initial blood chemistry demonstrated a rise in inflammatory markers—white cell counts 19.42 G/liter (normal upper limit 10 G/liter) and a C-reactive protein 7 mg/l (normal upper limit 12 mg/l), with a significant elevation in Lactates 20.4 mmol/l (normal upper limit 2 mmol/l).

The initial arterial blood gas analysis showed a metabolic acidosis with a pH of 7.09 and a alkaline reserve level of 5 mmol/L. Additionally, there was evidence of acute functional renal failure with a urea level of 18.1 mmol/L and a creatinine level of 226 µmol/L.

**Fig. 1:**
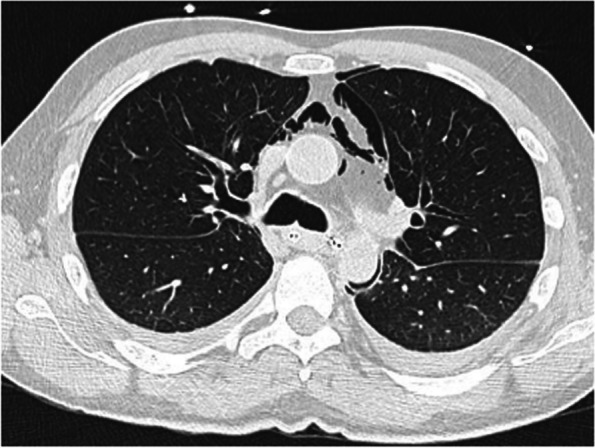
CT scan with contrast revealed a pneumomediastinum, right apical pneumothorax, bilateral pleural effusion, and cervical emphysema

The CT scan with contrast revealed a perforation of the lower third of the esophagus with pneumomediastinum, right apical pneumothorax, bilateral pleural effusion, and cervical emphysema, along with gastric distension (Fig. 1).

### Treatment

The patient's condition worsened quickly, requiring the initiation of NOREPINEPHRINE and subsequent oro-tracheal intubation. He was then transferred to the Saint Antoine Surgical Intensive Care Unit. An endoscopy performed upon his arrival confirmed a 5 mm esophageal perforation located 39 cm from the dental arches within a mucosa that appeared to have a circumferential esophagitis starting at 25 cm from the dental arches and extending to the cardia, which is situated 40 cm from the dental arches.

Initial treatment was broad-spectrum antibiotics using MEROPENEME, with a later addition of with a later addition of FLUCONAZOLE.

The patient underwent interventional endoscopy on his arrival, which revealed a 10 mm orifice located 40 cm from the dental arches within an esophagus where the mucosa seemed to have completely disappeared circumferentially over a ten- centimeter height, with only the uppermost 5 cm of the esophagus having a normal mucosa. There was also a Forrest grade 3 ulcer and a 15 mm subpapillary Forrest grade 2 ulcer. The fistula tract was catheterized and treated with the placement of three double-tailed pigtail stents, which were pushed towards the basithoracic right pleural collection. A naso-jejunal feeding tube was also inserted.

The patient remained febrile and in septic shock, requiring the reintroduction of Norepinephrine. A repeat CT scan on July 13th showed the pigtail drains still in place in the postero-inferior mediastinal collection, which had increased in size. A new digestive endoscopy was scheduled and performed on July 17th.

The surgical management involved abdominal esophageal suturing with right anterolateral thoracotomy for right pleural decortication. Left pleural drainage and jejunostomy for feeding were also performed. The esophageal suture was covered with an anterolateral tuberosity valve extending into the posterior infra-mediastinal space.

## Adverse events

The occurrence of external cardiac arrhythmias, including a sinus pause, prompted the necessity for an external cardiac pacemaker, which was successfully implanted on July 13th.

### Outcome and follow-up

Surgical repair was successful and he made a full recovery, discharged home within 1 month of initial presentation.

### Clinical benefits

The timing of the diagnosis and treatment plays a significant role in determining the outlook for patients with Boerhaave’s syndrome. Early diagnosis and surgical repair within 24 h of the rupture generally result in better outcomes.

Timely recognition, appropriate diagnostic evaluation, and immediate surgical intervention are crucial for the management of this life-threatening condition.

Overall, this case report emphasizes the importance of early diagnosis and aggressive management in improving outcomes for patients with Boerhaave’s syndrome.

## Discussion

Boerhaave’s syndrome, which was first described in 1724, is a rare and life- threatening condition caused by the spontaneous rupture of the esophagus due to acute barotrauma [6]. Diagnostic errors commonly occur because it is often mistaken for other conditions such as a perforated gastric ulcer, myocardial infarction, pulmonary embolism, pneumonia, dissecting aneurysm, and pancreatitis [7]. While radiological tests like a chest x-ray can help narrow down the possible causes (e.g., widened mediastinum and left-sided effusion), the definitive imaging method is a contrast CT scan as the signs are usually subtle [8].

The syndrome is more common in men, with a mean age between 40 and 60 years, in the majority of cases [8].

Boerhaave’s syndrome is characterized by a spontaneous rupture of the esophagus, typically caused by forceful vomiting. This condition is associated with significant morbidity and mortality, with a higher mortality rate compared to ruptures of other parts of the digestive tract. Early diagnosis and intervention are critical in the management of Boerhaave’s syndrome, as delayed treatment can lead to life- threatening complications such as mediastinitis and sepsis. Various studies have highlighted the importance of accurately distinguishing Boerhaave’s syndrome from other conditions with similar presentations, such as Mallory-Weiss syndrome.

Many published case reports comment on Mackler’s triad of vomiting, chest pain and emphysema, as key clinical signs and symptoms [9]. Our case supports these criteria.

Others suggest acute symptoms and signs to include hemodynamic instability and a Hammer sign on auscultation (crackling on chest auscultation— pneumomediastinum) [10].

Recognizing the various signs and symptoms of this syndrome is crucial for physicians to consider it as a possible diagnosis. This becomes especially important when initial treatment approaches yield inconsistent results in symptom control.

Boerhaave’s syndrome has a high mortality rate, with estimates ranging from 20 to 40%. However, early diagnosis and prompt surgical intervention significantly improve the patient's chances of survival. Therefore, it is essential for healthcare professionals to consider Boerhaave’s syndrome in patients presenting with acute chest pain and a history of vomiting.

Timely recognition, appropriate diagnostic evaluation, and immediate surgical intervention are crucial for the management of this life-threatening condition. Overall, this case report emphasizes the importance of early diagnosis and aggressive management in improving outcomes for patients with Boerhaave’s syndrome.

Boerhaave’s syndrome is often misdiagnosed initially due to its resemblance to other common conditions, but a careful history and thorough evaluation can help reveal key clinical features that are characteristic of Boerhaave’s syndrome. Some of the common presenting symptoms of Boerhaave’s syndrome include vomiting, chest pain, dyspnea, and subcutaneous emphysema. Diagnostic imaging, particularly chest X-rays, can aid in the identification of radiographic signs, such as pneumomediastinum and pneumoperitoneum. It is essential for healthcare providers to be aware of these clinical features and radiographic findings in order to make an accurate diagnosis and initiate timely management.

Once Boerhaave’s syndrome is suspected, further diagnostic modalities such as computed tomography can be used to assess the severity of the condition and identify complications such as mediastinitis. Management of Boerhaave’s syndrome typically involves surgical intervention, with primary repair of the esophageal rupture being the mainstay of treatment. However, with advancements in the field of endoscopy, multiple endoscopic approaches have been successfully used in the management of Boerhaave’s syndrome [11].

These include endoscopic stenting, fibrin glue injection, and endoscopic vacuum therapy. These endoscopic techniques offer a less invasive alternative to surgery and have shown promising results in select cases of Boerhaave’s syndrome.

Shaker et al. describe a decline in mortality rate with early diagnosis and surgical treatment, within 24 h of presentation from 40% to 6.7% [12]. Surgical repair is seen as the gold standard, with significant reduced mortality from early intervention within the first 24 h from presentation.

In our case, the patient underwent two attempts with interventional endoscopy, before abdominal esophageal suturing.

Furthermore, distinguishing Boerhaave’s syndrome from Mallory-Weiss syndrome is crucial in order to provide appropriate treatment and avoid potential complications. In cases where the initial diagnosis is uncertain, endoscopy can be used to identify the source of bleeding in Mallory-Weiss syndrome, as radiological modalities are not effective in detecting it [13].

In summary, it is crucial to accurately diagnose Boerhaave’s syndrome in order to provide timely and appropriate treatment. A thorough evaluation, including a careful history and diagnostic imaging such as chest X-rays and computed tomography, can help in accurately diagnosing this rare condition. Healthcare providers should be aware of the characteristic clinical features and radiographic findings associated with Boerhaave’s syndrome. Additionally, it is important to differentiate Boerhaave’s syndrome from Mallory-Weiss syndrome to ensure proper treatment and avoid complications. If the initial diagnosis is uncertain, endoscopy can be used to differentiate between the two conditions and guide appropriate treatment. Accurate diagnosis of Boerhaave’s syndrome is crucial due to its potential life-threatening nature. Prompt recognition and early management of Boerhaave’s syndrome can greatly impact patient outcomes.

Overall, accurate diagnosis and prompt management play a critical role in the treatment of Boerhaave’s syndrome [14].

## Conclusion

Boerhaave’s syndrome is a rare condition characterized by a spontaneous rupture of the esophagus, typically in its lower portion. Timely identification and treatment are crucial for improved prognosis. Preferred management involves surgical procedures such as mediastinal and chest drainage, along with esophageal repair, resection, or exclusion, offering the best chances of survival.

This case report highlights the importance of considering Boerhaave’s syndrome in patients presenting with acute chest pain and a history of vomiting or retching.

Prompt recognition, appropriate diagnostic evaluation, and immediate surgical intervention are crucial for the management of this life-threatening condition.

Increased awareness among healthcare professionals can lead to earlier diagnosis and potentially better outcomes in patients with Boerhave’s syndrome.

## Data Availability

No datasets were generated or analysed during the current study.
